# Editorial: Regulation of osteoclast differentiation in autoimmune and inflammatory diseases

**DOI:** 10.3389/fimmu.2022.1125763

**Published:** 2023-01-05

**Authors:** Sun-Kyeong Lee, Antonio Inforzato, Cristina Sobacchi

**Affiliations:** ^1^ University of Connecticut (UCONN) Center on Aging, University of Connecticut (UCONN) Health, Farmington, NM, United States; ^2^ IRCCS Humanitas Research Center, Rozzano, Italy; ^3^ Department of Biomedical Sciences, Humanitas University, Pieve Emanuele, Italy; ^4^ CNR-IRGB, Milan Unit, Milan, Italy

**Keywords:** osteoclast, inflammation, cytokines, gut-immune-bone axis, therapy, BMPs, notch signaling, RANKL

Bone remodeling is a dynamic and tightly regulated process that relies on a fine balance between deposition and resorption of the unique mineralized extracellular matrix (ECM) of the musculoskeletal system. Bone-resorbing osteoclasts are multinucleated cells that originate primarily from bone-marrow myeloid and, in specific conditions, extramedullary erythromyeloid progenitors through the combined action of Macrophage Colony-Stimulating Factor (M-CSF) and Receptor Activator of Nuclear Factor κ B-Ligand (RANK-L). In addition to these canonical osteoclastogenic stimuli, differentiation, activation, and recycling of osteoclasts are sustained by other soluble, ECM, and cell-borne factors, including bone and immune cell-derived inflammatory mediators (e.g., TNF-α, IL-1β, IL-6, IL-17, IL-23). When in excess over their physiological concentration, these might trigger and maintain a chronic status of local and/or systemic inflammation that eventually leads to hyperosteoclastogenesis and osteolysis, both of which are common traits of autoimmune (e.g., rheumatoid arthritis, inflammatory bowel disease) and inflammatory/infectious (e.g., osteomyelitis and periodontitis) diseases of the bone. Despite impressive progress in osteoimmunology, the cellular and molecular mechanisms underpinning the disregulation of osteoclastogenesis in bone pathology are largely unknown.

In the study by Kovacs et al., the differentiation of osteoclasts from human peripheral blood mononuclear cells (PBMCs) was tracked *in vitro* through proteomics. Increased expression of mitochondrial, ribosomal, and basolateral membrane proteins was observed in osteoclasts and preosteoclasts as compared to monocytes, with a concomitant decline in components of secretory pathways (e.g., reduced cytokine release and MHC trafficking) and immune processes (e.g., inflammation, migration). Consistent with a transition from an immune to a bone-resorbing type of cell, osteoclasts and preosteoclasts were biased towards carbohydrates as a preferential source of energy for the active synthesis of bone-resorbing enzymes (e.g., cathepsin K and B, lysosomal acid phosphatase) and for the acidification of resorption lacunae (e.g., by V-ATPase). Interestingly, this trajectory was partially reversed in osteoclasts obtained from the PBMCs of rheumatoid (RA) and psoriatic arthritis (PsA) patients. Indeed, these cells retained some of their original immunological characteristics (e.g., expression of MHC I and II), and had reduced metabolic polarization when compared to their healthy counterparts. Based on these findings, it was proposed that the inflammatory milieu of RA and PsA might favor the formation of “immune-like” osteoclasts that might act as inflammatory agents more than bone-resorbing cells in contributing to osteolysis.

Taking advantage of a lineage tracing strategy and an animal model of collagen-induced arthritis (CIA), Filipovic et al. addressed the effect of arthritis-associated inflammation on the transcriptome of osteoclast progenitors (OCPs). These OCPs were classified based on the expression of CCR2, namely circulatory-like CCR2^hi^, which are rich in CCR2, have high osteoclastogenic potential, and expand in arthritis, and bone marrow-resident CCR2^lo^ OCPs, which are poor in CCR2, are less mature than CCR2^hi^, and are likely to participate in bone homeostasis. These two subsets, which possibly reflect different phases of the differentiation trajectory proposed by Kovacs et al., have distinctive profiles of gene expression, with CCR2^hi^ enriched in pathways for osteoclast differentiation, chemokine signaling, and inflammation (e.g., *Fcgr1*, *Socs3*, *Irf7*, *Itgam*), and CCR2^lo^ expressing higher levels of biosynthetic genes (e.g., *Gnl3*, *Dctd*). Importantly, *F11r*, *Cd38*, and *Lrg1* were proposed as arthritis-specific genes, based on the observation that their expression in the CCR2^hi^ subset, both as mRNAs and proteins, positively correlated with disease severity. These molecules are involved in migration, adhesion, and homing, which suggests that circulating CCR2^hi^ cells are recruited to the arthritic bone, where they might contribute to bone erosion. It is therefore conceived that *F11r*, *Cd38*, and *Lrg1* could be useful not only as disease markers but also as molecular targets for pharmacological interventions in arthritis.

In this regard, novel perspectives on the pharmacology of bone loss-associated diseases were opened by Cao et al., who reported on the inhibitory effects of Sec-O-glucosylhamaudol (SOG) on osteoclastogenesis. SOG is a flavonoid derived from the root of *Saposhnikoviae divaricata* that is endowed with analgesic, anti-inflammatory, and 5-lipoxygenase (5-LO) inhibiting properties. Based on the number of TRAP-positive multinucleated cells, formation of resorption pits and F-actin rings, and expression of osteoclast-specific genes (including the transcription factors *NFATc1* and *c-Fos)*, this compound effectively inhibited RANK-L-induced osteoclastogenesis *in vitro* in a setting where murine bone marrow macrophages were used as osteoclast precursors. SOG exerted its anti-osteoclastogenic effect through at least two independent mechanisms involving inhibition of: *i)* 5-LO, which that catalyzes the synthesis of leukotrienes, and is involved in inflammatory disorders of the bone, and *ii)* AKT-dependent inactivation of GSK3β, which promotes nuclear localization and transcriptional activity of NFATc1. Of note, this flavonoid had bone-protective effects in an inflammatory (LPS) murine model of bone loss, suggesting potential therapeutic applications in osteolytic diseases.

Since the discovery of the involvement of the immune system in osteoporosis, which set the field of “immunoporosis”, the pharmacological modulation of specific adaptive immune cell subsets (i.e., Bregs, Tregs and Th17 cells) has gained attention as a bone anabolic therapy for osteoporosis. In particular, manipulation of the gut microbiota through probiotics administration has been demonstrated to improve bone health through a gut-immune-bone axis. In this regard, Sapra et al. elucidated the immunoporotic potential of the gram-positive, anaerobic bacterium *Bifidobacterium longum* (BL) in a mouse model of postmenopausal osteoporosis. This study demonstrated that BL induced the formation of a CD19^+^CD1d^+^CD5^+^IL10^+^ Breg population with increased capacity to suppress osteoclast formation, both from murine and human precursors, and to modulate CD4^+^Foxp3^+^IL-10^+^ Treg and CD4^+^Rorγt^+^IL-17^+^ Th17 differentiation *in vitro*. Accordingly, *in vivo*, a higher frequency of Bregs and Tregs and a lower frequency of Th17 cells were found in the bone marrow and spleen of BL-treated ovariectomized (ovx) mice, along with higher serum levels of antiosteoclastogenic cytokines, such as IL-10 and IFN-γ (hallmark of Bregs and Tregs), and lower levels of pro-osteoclastogenic cytokines, such as TNF-α, IL-6 and IL-17 (hallmark of Th17 cells). Overall, this resulted in preserved bone microarchitecture and improved bone mineral density and mechanical strength in BL-supplemented ovx mice. The bone-sparing and immunomodulatory ability of this specific probiotics strain seems promising for exploitation in human bone loss conditions. Future research will identify the specific metabolites that are responsible for this effect.

In the field of chronic inflammatory diseases, therapeutic strategies have been developed to inhibit Notch signaling, known to play an essential role in T cell and marginal zone B cell lineage commitment. Myeloid cells are important effectors of inflammation, comprising macrophages, osteoclasts, and dendritic (MOD) cells, all originating from a common MOD progenitor (MODP). However, our current understanding of the role of the Notch pathway in the fate decision in this lineage is incomplete. Filipovic et al. isolated the CD45^+^Ly6G^-^CD3^-^B220^-^NK1.1^-^CD11b^-/lo^CD115^+^ trilineage progenitors from the bone marrow of conditional inducible CX3CR1CreERT2 mice and used these MODPs to achieve either Notch signaling overexpression or inhibition *in vitro* and to investigate the respective impact on osteoclast, macrophage, and dendritic cell formation and function. They found that Notch signal modulation affected all three lineages. Specifically, Notch activation suppressed osteoclastogenesis, while its silencing enhanced osteoclast formation and function, and this effect was even more pronounced in inflammatory conditions. Macrophage and dendritic cell formation did not change upon Notch modulation. Macrophage phagocytosis was enhanced both after Notch pathway activation and inhibition, whereas antigen presentation by dendritic cells in inflammatory conditions was downregulated upon pathway activation. This work highlighted cell-autonomous responses to Notch stimulation. Owing to the complexity of this ligand/receptor system and the influence exerted on it by environmental factors, the described mechanisms deserve attention when considering the possibility of targeting this pathway pharmacologically.

Finally, Bordukalo-Nikšic et al. provided an overview of the role of bone morphogenetic proteins (BMPs) in bone biology, underlying that these multi-functional growth factors not only stimulate osteoblast differentiation, mineralization, and survival (e.g., BMP2, -4, -5, -6, -7 and -9), but are also important for osteoblast-osteoclast crosstalk (e.g., BMP6) and for increasing osteoclast formation (e.g., BMP2, -5, -6 and -7) and resorption (e.g., BMP4 and -7). The multifaceted activity of BMPs was also apparent in the physiological process of fracture healing, as well as in inflammatory disorders of the bone and joints, such as RA and Ankylosing Spondylitis (AS). It also explains the side effects of therapeutic BMP devices in clinical use or clinical trial, pointing to the need for improved therapeutics to more effectively exploit their potential as bone-healing agents.

Overall, the works collected in this Research Topic shed new light on the cellular and molecular mechanisms leading to the disregulation of osteoclastogenesis in bone pathology and identify potential strategies to be further pursued for translational application.

## Author contributions

All authors listed have made a substantial, direct, and intellectual contribution to the work and approved it for publication.

